# Chimeric synthetic reference standards enable cross-validation of positive and negative controls in SARS-CoV-2 molecular tests

**DOI:** 10.1038/s41598-021-81760-0

**Published:** 2021-01-29

**Authors:** Bindu Swapna Madala, Andre L. M. Reis, Ira W. Deveson, William Rawlinson, Tim R. Mercer

**Affiliations:** 1 Genomics and Epigenetics Theme, Garvan Institute of Medical Research, Sydney, NSW Australia; 2 Kinghorn Centre for Clinical Genomics, Garvan Institute of Medical Research, Sydney, NSW Australia; 3 St Vincent’s Clinical School, University of New South Wales, Sydney, NSW Australia; 4 School of Medical Sciences, Women’s & Children’s, and School of Biotechnology and Biomolecular Sciences, University of New South Wales, Sydney, NSW Australia; 5 Australian Institute of Bioengineering and Nanotechnology, University of Queensland, Brisbane, QLD Australia

**Keywords:** Diagnostic markers, Viral infection

## Abstract

DNA synthesis in vitro has enabled the rapid production of reference standards. These are used as controls, and allow measurement and improvement of the accuracy and quality of diagnostic tests. Current reference standards typically represent target genetic material, and act only as positive controls to assess test sensitivity. However, negative controls are also required to evaluate test specificity. Using a pair of chimeric A/B RNA standards, this allowed incorporation of positive and negative controls into diagnostic testing for the Severe Acute Respiratory Syndrome Coronavirus-2 (SARS-CoV-2). The chimeric standards constituted target regions for RT-PCR primer/probe sets that are joined in tandem across two separate synthetic molecules. Accordingly, a target region that is present in standard A provides a positive control, whilst being absent in standard B, thereby providing a negative control. This design enables cross-validation of positive and negative controls between the paired standards in the same reaction, with identical conditions. This enables control and test failures to be distinguished, increasing confidence in the accuracy of results. The chimeric A/B standards were assessed using the US Centres for Disease Control real-time RT-PCR protocol, and showed results congruent with other commercial controls in detecting SARS-CoV-2 in patient samples. This chimeric reference standard design approach offers extensive flexibility, allowing representation of diverse genetic features and distantly related sequences, even from different organisms.

## Introduction

Reference standards are required to validate the performance of any diagnostic test^1^. The recent advent of DNA synthesis enables the rapid development of reference standards as synthetic constructs representing target genetic material. These can be used as positive controls to assess sensitivity of the molecular test undergoing evaluation. However, separate negative controls, without the target sequences, are also required to ensure the specificity of the diagnostic test. Reference standards must be validated and proven fit-for-purpose before used in diagnostic tests. In the case of RNA standards, the synthetic controls can also undergo degradation over time, and can be contaminated, confounding the interpretation of test results. However, this failure of either positive or negative controls is difficult to distinguish from the failure of the diagnostic test itself. For example, if the test returns a negative result from the positive control, it could be because (i) the test failed, (2) the reference control failed or (3) a technical issue with the testing platform. This leads to delays in diagnosis, missed diagnoses and invalidation of correct test results.

The use of in vitro synthesis of RNA and DNA standards allows flexibility in control design and tailoring of controls to the diagnostic test and targets. Here, we propose a new design strategy for reference standards that uses matched chimeric synthetic standards in accordance with the principle of A/B testing. In this design, all the target sequences of a molecular test are retrieved and split between groups A and B, which are then joined in tandem to form single chimeric sequences A and B. This means that for each target used in the molecular test, standard A would act as positive control, while standard B would act as negative control, or vice-versa. Furthermore, the equally partitioning of target sites between standards A and B enables cross-validation of positive and negative controls, increasing the confidence in test results. Among the benefits of this design, a chimera allows concurrent testing of disparate target regions of a single pathogen or even different organisms and splitting targets between standards A and B enables control cross-validation, facilitating the distinction of control failure from test failure or success.

The recent emergence of the SARS-CoV-2 pandemic has required widespread diagnostic testing for active virus infections. Testing predominantly uses real-time reverse-transcriptase polymerase chain reaction (real-time RT-PCR)-based assays^2–4^. The World Health Organisation (WHO) published seven diagnostic testing protocols for detection of SARS-CoV-2 that have been rapidly adopted worldwide, with over 20 million molecular tests performed globally by mid-2020^5,6^. These tests typically employ multiple primer pairs homologous with SARS-CoV-2 genes *E, N, Orf 1a/1b* and *RdRp*^7–9^. Diagnosis is considered positive if all targets are amplified or presumptive positive if some but not all targets are detected. In addition, some tests contain primer pairs also targeting human genes as internal positive controls to ensure sample quality.

We used a pair of chimeric A/B standards for the WHO-endorsed real-time RT-PCR tests to demonstrate the utility of the chimeric A/B approach in designing reference test standards. Each standard included regions of the coronavirus genome (SARS-CoV-2) that are targeted by published primer pairs. As a result, it is compatible with endorsed diagnostic tests licensed globally. We compared the performance of the synthetic controls to other reference materials and patient samples, and demonstrated how the two synthetic RNA standards can be used to validate the standard real-time RT-PCR test (CDC), and also considered the utility of these standards in other assays^10^.

## Results

### Design of chimeric RNA reference standards for SARS-CoV-2

DNA synthesis enables rapid and flexible assembly of reference standards, including sequences not present in natural organisms. This allows sequences from different genome regions to be aggregated to address specific requirements in a diagnostic assay. To demonstrate this approach, we designed synthetic reference sequences that encompass the primer binding sites of all WHO-published real-time RT-PCR tests.

**Figure 1:**
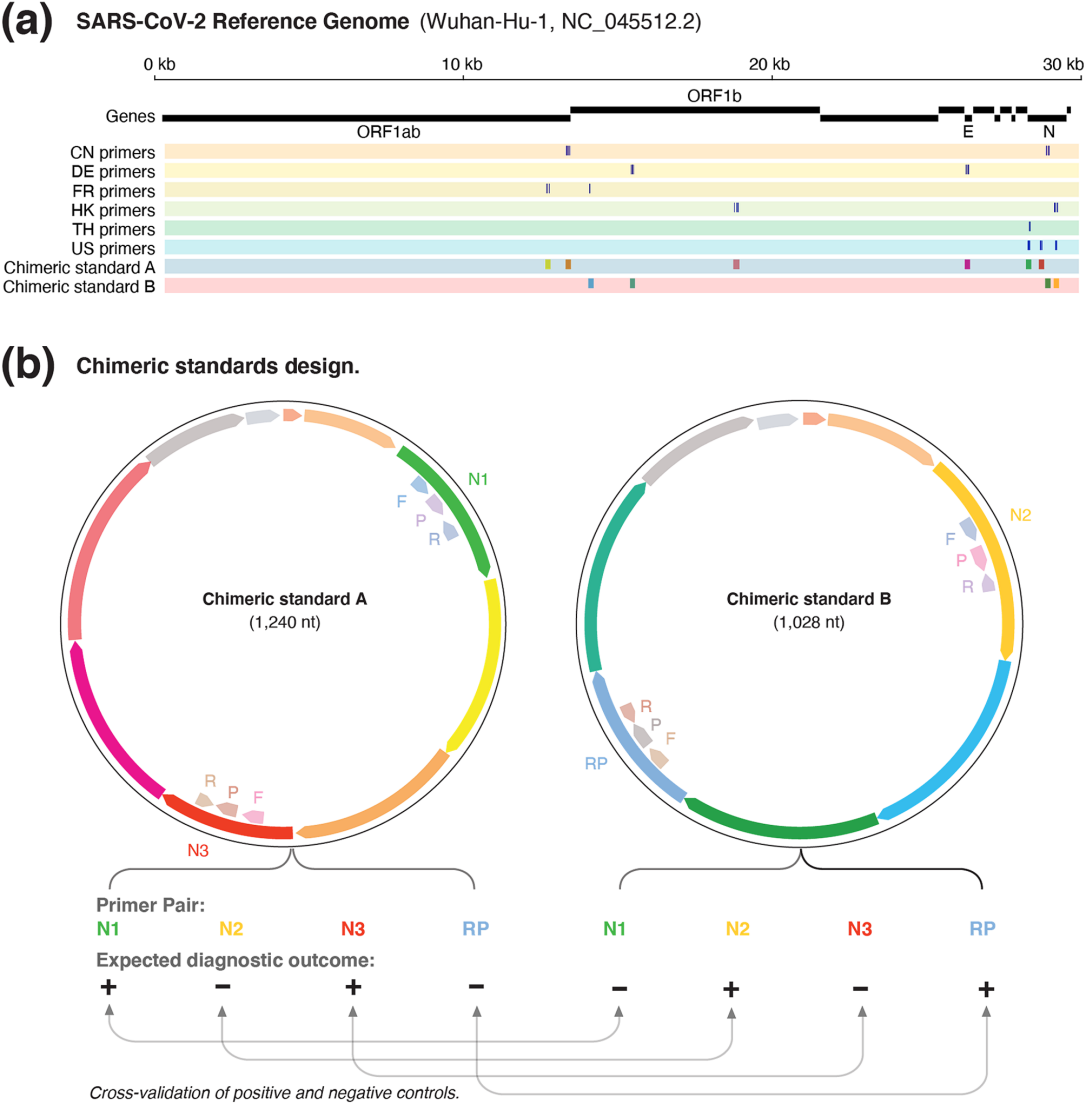
Design of chimeric controls for SARS-CoV-2. (**a**) View of the SARS-CoV-2 genome showing genes targeted by WHO-published real-time RT-PCR primer/probe sets (ORF1a, ORF1b, E and N genes). Specific binding sites targeted by different countries are shown in individual tracks (CN = China, DE = Germany, FR = France, HK = Hong Kong, TH = Thailand and US = United States). Targeted regions encompassing several primer/probe sets were selected and exclusively partitioned between chimeric A/B standards. The expected amplicons for the CDC test are shown in the last track. (**b**) The different targeted regions for standards A and B were shuffled and joined together to form chimeric sequences. The paired design of chimeric A/B standards, where a target in A is absent in B (and vice versa), enables the synthetic RNA transcripts to simultaneously act as positive and negative controls for the real-time RT-PCR primer/probe sets (F = forward primer, R = reverse primer and P = probe). This enables internal cross validation of positive and negative controls between standards A and B. The vector backbone was omitted from the representation of the chimeric A/B standards. The colours for targeted regions in the chimeric A/B standards are the same between figures (**a**) and (**b**).

We first retrieved the SARS-CoV-2 genome sequence (isolate Wuhan-Hu-1, NC_045512.2), as well as the primer sequences published by the World Health Organisation (WHO) for China, Hong Kong, Thailand, United States (CDC), Germany and France (Fig. 1a). Each available real-time RT-PCR test typically comprises 2–3 primer pairs that target different regions of the SARS-CoV-2 genome (see Supplementary Table 1). We then aligned the primer pairs to the SARS-CoV-2 genome and identified the coordinates of the amplicons, which were then retrieved along with an additional 30 nucleotides (nt) on either flanking side (Fig. 1a).

We next organised these sequences across two different controls (termed chimeric A/B standards). We partitioned the different targeted regions used by each country into two independent groups and then assembled the regions in tandem (Fig. 1b). A fragment of the human RNase P gene (RP), which is used as a positive human control, was also added to the chimeric standard B.

An additional unique control sequence (UCS) was also included at the 5′ region of each standard to enable the unique detection of the standards if required. Each standard sequence was then preceded by a T7 promoter to enable in vitro transcription, and followed by a poly-A tract (30nt length) and a restriction site (EcoR1) to enable vector linearization (see Supplementary Table 2).

The two distinct chimeric A/B standard sequences were then synthesised and cloned into pMK vector backbones (see “Methods”). We then linearized the plasmids and performed in vitro transcription to produce the synthetic RNA standards (Fig. S1). The resulting RNA transcript products were then purified and validated (see “Methods” and Supplementary Methods for detailed protocol).

### Validation of chimeric RNA standards to alternative reference controls

We next validated the chimeric A/B standards by comparison to alternative reference controls. We first performed real-time RT-PCR test using the established CDC primers and protocol^10^. Specifically, this employs CDC primers (IDT) *N1, N2* and *N3* targeting the N gene from SARS-CoV-2 and the human *RNase P* gene. Chimeric standard A includes regions of the *N1* and *N3* targets, while chimeric standard B includes regions of the *N2* and *RP* targets. This allows for cross-validation between the chimeric A/B standards, since the standards alternatively act as positive and negative controls to each primer/probe set in the real-time RT-PCR test.

**Figure 2:**
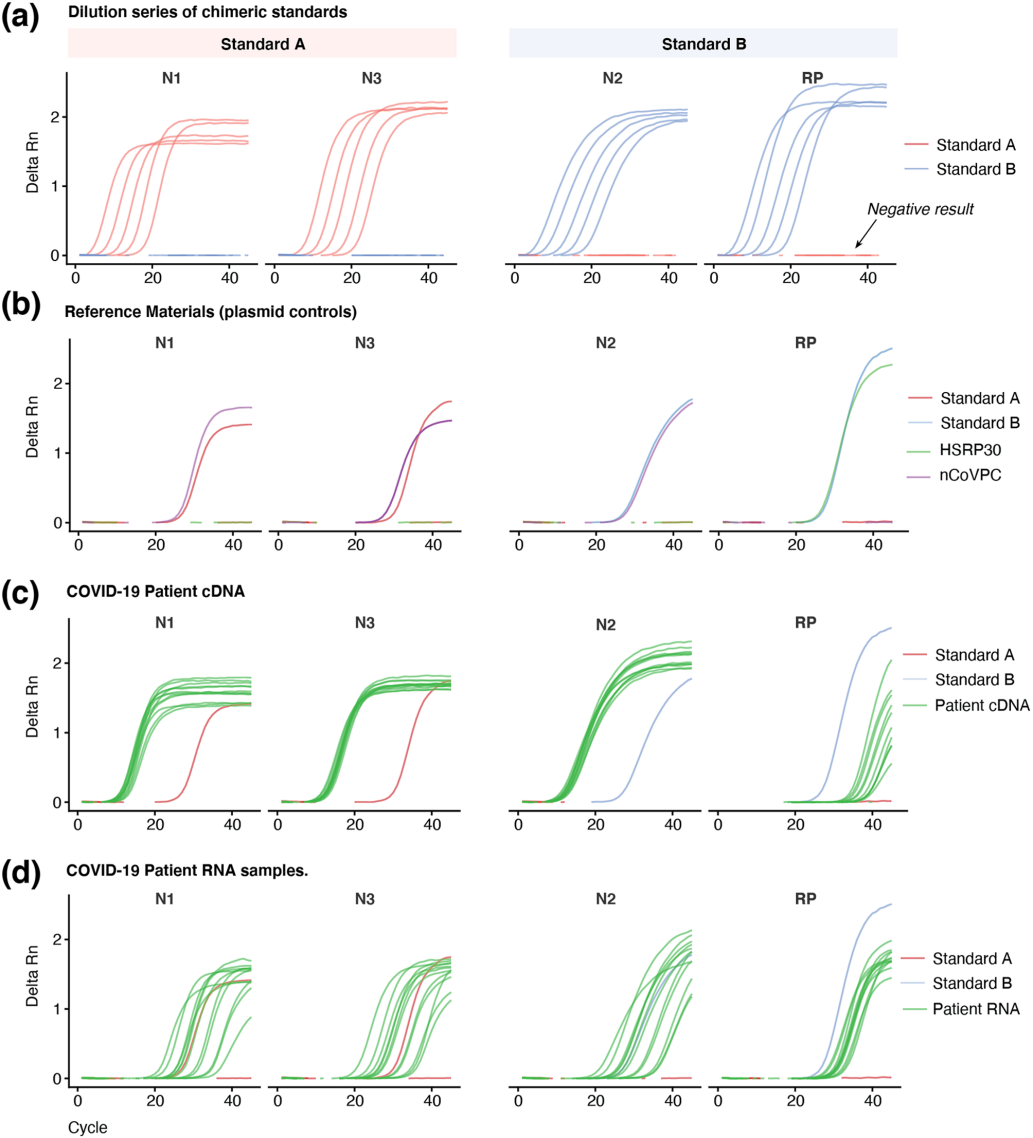
Real-time RT-PCR validation of chimeric A/B sandards. Amplification curves for genes targeted in the CDC real-time RT-PCR test for SARS-CoV-2 (N1, N2, N3 and RP). (**a**) Dilution series showing amplification curves for standards A (red line) and B (blue line). (**b**) Amplification curves comparing the chimeric A/B standards with IDT positive (nCoVPC, purple line) and negative controls (HSRP30, green line). (**c**, **d**) Amplification curves comparing the chimeric A/B standards with positive COVID-19 cDNA and RNA patient samples (green line, n = 12).

The real-time RT-PCR was initially performed on the chimeric controls alone. We prepared tenfold dilutions for each control, starting at 3.96 × 10^8^ copies/μl for A and 4.22 × 10^8^ copies/μl for B (see “Methods”). As anticipated, in the reactions containing standard A, *N1* and *N3* primers returned positive results, while N2 and RP were undetected (Fig. 2a). In contrast, in reactions containing standard B, *N2* and *RP* primers returned positive results, while *N1* and *N3* were undetected (Fig. 2a). In the real-time RT-PCR reactions with positive results, there is an average increase in Ct values of 3.47 (sd = 0.34) for a tenfold dilution. These results show that the chimeric controls enable positive and negative cross-validation of the published CDC real-time RT-PCR test for SARS-CoV-2.

We next performed the real-time RT-PCR test including commercial controls (Integrated DNA Technologies Ltd.) for comparison with the chimeric A/B standards. The positive and negative commercial controls are provided as separate plasmids. The positive control (2019-nCoV_N_Positive Control) contains the complete nucleocapsid gene for SARS-CoV-2, while the negative control (Hs_RPP30) contains a fragment of the human RNase P gene.

Therefore, IDT positive control, 2019-nCoV_N_Positive Control, should detect *N1, N2* and *N3*, but not *RP*, while, IDT negative control, *Hs_RPP30,* should only detect RP. We diluted each plasmid to 4,000 copies/μl along with the chimeric A/B standards and performed the real-time RT-PCR test with the CDC primers and probes. The IDT controls worked as expected and the chimeric A/B standards achieved similar results (Fig. 2b). For each of the target genes, The Ct values were comparable between the IDT controls (N1 = 26; N2 = 28; N3 = 28; RP = 27) and the chimeric A/B standards (N1 = 27; N2 = 27; N3 = 30; RP = 27).

### Comparison of chimeric RNA standards COVID-19 patient samples

To validate the commutability of the chimeric A/B standards, we compared their performance in amplifying genomes from 12 COVID patient samples that had been independently diagnosed and sequenced (see "Methods"). These samples were available as raw total RNA containing the viral genome and cDNA, where the viral genome was previously amplified. Therefore, the real-time RT-PCR test with CDC primers should yield positive results for all target genes (*N1, N2, N3* and *RP*) in patient RNA samples and primarily positive results for *N1, N2* and *N3* in patient cDNA samples.

We diluted patient cDNA samples so that on average reactions had 2.79 ng (sd = 0.35) of input DNA. We did not measure the concentration of patient RNA samples due to insufficient starting materials. The real-time RT-PCR amplification results showed that both chimeric A/B standards and patient samples were diagnosed as expected. Target genes *N1, N2* and *N3* were amplified in both patient cDNA (avg Ct; N1 = 12 ± 0.72, N2 = 12 ± 0.70, N3 = 13 ± 0.69) and patient RNA (avg Ct; N1 = 29 ± 4.85, N2 = 29 ± 4.92, N3 = 29 ± 4.89) samples, with the former achieving significantly lower and less variable Ct values (Fig. 2c,d). However, for the *RP* target gene, which is a positive control for human samples, the Ct value for patient cDNA was significantly higher, since those samples are depleted of human material (37 ± 1.83 and 31 ± 1.54, respectively).

**Figure 3:**
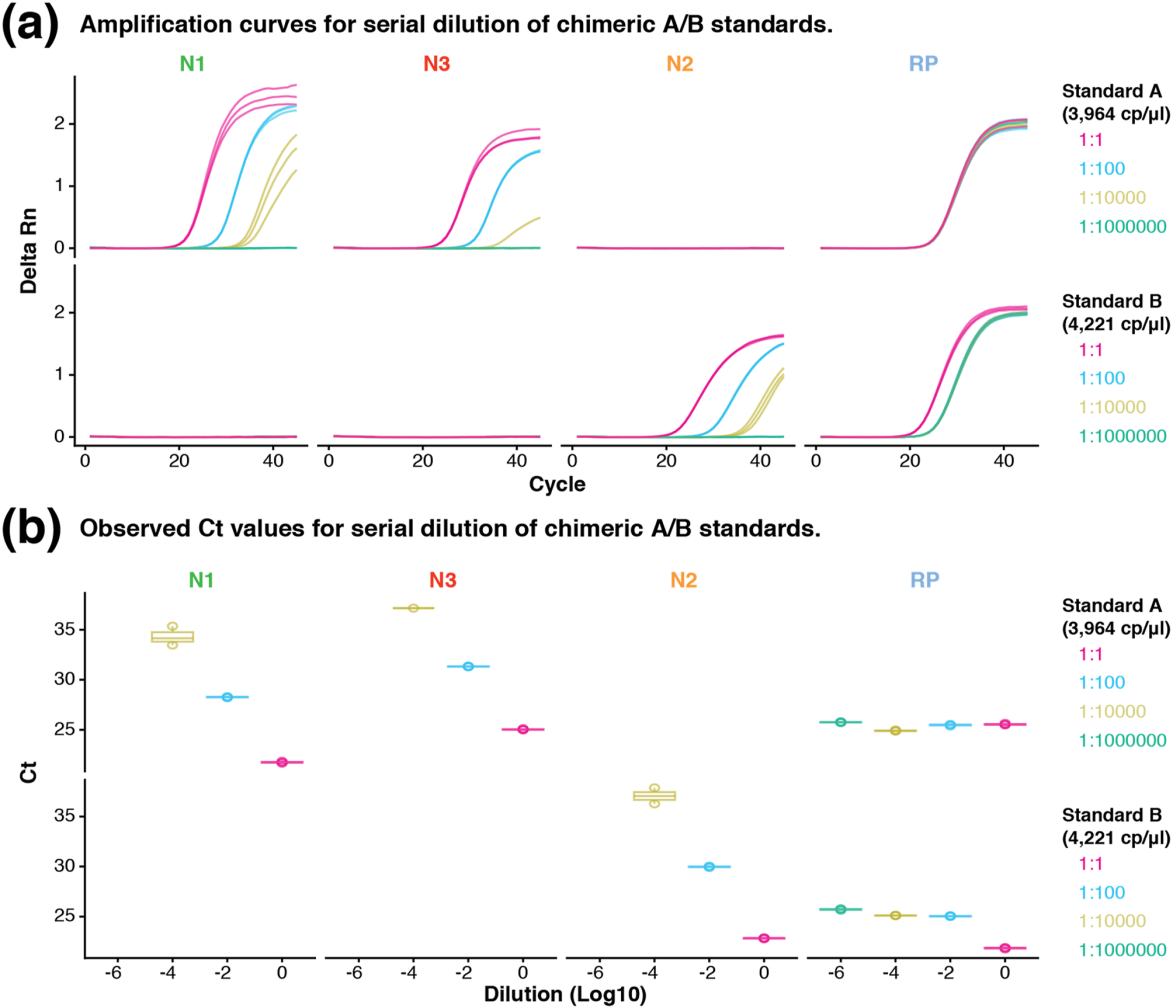
Limit of detection of chimeric A/B standards. (**a**) Amplification curves of targets *N1, N2, N3* and *RP* in 100-fold serial dilutions of standards A and B against the human universal RNA, with three technical replicates. (**b**) Observed Ct values for targets N1, N2, N3 and RP at different dilutions of standards A and B (10^0^, 10^–2^, 10^–4^ and 10^–6^), across three technical replicates.

To determine the limit of detection (LoD) for the chimeric A/B standards, we performed 100-fold serial dilutions with three technical replicates. As a baseline, we spiked the standards A (3964 copies/μl) and B (4221 copies/μl) into separate background samples consisting of the human universal RNA (100 ng). We then performed real-time RT-PCR using the CDC protocol (see "Methods"). As a result, we detected both standards A and B until 10^–4^ dilution, which corresponds to approximate LoD of 0.39 and 0.42 copies/μl, respectively (Fig. 3a). As a positive control, the RP primer targeting the human *RNase P* gene were successfully detected in all tested dilutions, for both standards A and B (Fig. 3b). Interestingly, N1 primers appear to be more efficient than N3 primers in estimating the LoD for standard A, since the Ct values for N1 (10^0^ = 21.75 ± 0.09, 10^–2^ = 28.26 ± 0.04 and 10^–4^ = 34.33 ± 0.96) are significantly lower than N3 (10^0^ = 25.04 ± 0.06, 10^–2^ = 31.33 ± 0.04 and 10^–4^ = 37.16), in every dilution, across replicates (Fig. 3b).

## Discussion

The advent of routine DNA synthesis has enabled rapid provision of synthetic reference standards that can be used to validate the accuracy of diagnostic tests. The synthesis of DNA provides a flexible platform to manufacture different reference standards, including non-natural designs. In this case, a single standard can be designed to contain distant genomic regions or even sequences from different organisms. This allows multiple sequences of interest to be included and organised within a single chimeric standard according to the specific requirements of a diagnostic assay. In this study, we use this approach to generate chimeric RNA transcripts that encode different SARS-CoV-2 genomic regions targeted by WHO sanctioned molecular diagnostic tests.

Furthermore, we show how chimeric standards enables matched A/B testing of each primer/probe set used in the real-time RT-PCR. Because the target regions are exclusively distributed between the A/B standards, each inevitably functions as an independent positive or negative control for a given primer/probe set. Additionally, the balanced distribution of target regions for each WHO sanctioned test between A/B standards, ensures cross-validation of positive and negative controls. This improves the confidence in the results of the controls and prevents a test failure being confused with a control failure.

Within this study we successfully validated the chimeric A/B standards using the CDC real-time RT-PCR test for SARS-CoV-2 detection^10^. However, the chimeric A/B standards also contain the target sites in accordance to official guidelines for molecular testing in China, Thailand, Hong Kong, Germany and France, besides those of the United States. This means that the chimeric A/B standards can not only be used as test controls in each of those countries individually, but also provide a common reference to compare test results and the efficiency of their different primer/probe sets.

The chimeric reference standards can incorporate additional sequences. The flexibility offered by DNA synthesis enables the creative design of reference standards that can address the specific requirements of diagnostic tests. Within this study, we use chimeric design to incorporate positive and negative controls into single reference standards. However, the chimeric controls could also be designed to incorporate sequences from other genomes. For example, the inclusion of related gene sequences from other coronaviruses could enable the specificity of diagnostic assays to be simultaneously evaluated. This would ensure that qRT-PCR tests are specific for SARS-CoV-2, even in the presence of related sequences from other circulating coronaviruses that cause the common cold.

Chimeric designs reference standards could also be used in diagnostic applications beyond SARS-CoV-2. For instance, a single synthetic control could represent multiple genetic features, such as mutations, different haplotype blocks or pan-genome controls that incorporates sequences for different organisms. A single reference standard could include multiple microbe sequences, cancer mutations or expressed gene signatures. The flexibility offered the chimeric A/B approach provides a new approach to the development of reference standards tailored for the specific requirements of a diagnostic assay.

## Materials and methods

### Design of chimeric controls

We first retrieved from the SARS-CoV-2 reference genome sequence (isolate WuHan-Hu-1, NC_045512.2). We then aligned all RT-PCR primer pairs retrieved from the WHO website (accessed March 17th 2020). https://www.who.int/docs/default-source/coronaviruse/whoinhouseassays.pdf.

Expected amplified sequenced between primer pairs were generated using the In silico* PCR* tool from the UCSC Genome Browser for SARS-CoV-2. https://genome.ucsc.edu/cgi-bin/hgTracks?db=wuhCor1&lastVirtModeType=default&lastVirtModeExtraState=&virtModeType=default&virtMode=0&nonVirtPosition=&position=NC_045512v2%3A1%2D29903&hgsid=981801555_dhlOJ69K0oZXeDPZMPT9IQdZ5uBR.

We also included an additional 30nt upstream and downstream, and any overlapping sequences were collapsed into a single sequence. The amplicon sequences where then manually distributed across two standards. Additional control sequences comprising randomly synthetic nucleotide sequences (i.e. no homology > 21nt to natural sequences) to the upstream 5′ region of the control. The individual sequences were then conjoined together to form a pair of contiguous sequences. The control sequences were then submitted for synthetic by third-party vendor (ThermoFisher, GeneArt).

### Synthesis and preparation of chimeric standards

The A/B standard sequences were synthesized within pMK vectors by a commercial vendor (ThermoFisher, GeneArt). Following receipt, plasmid sequences were independently confirmed by Sanger sequencing. The plasmids containing the A/B standard sequences were then resuspended in 50 μl nuclease free water and transformed in *E. coli* as per manufacturer’s protocol (α-Select Competent Cells, Bioline, Australia). The transformed cells were grown overnight (37 °C) in LB agar plate containing Kanamycin (100 μg/ml), after which colonies were selected and further cultured overnight (37 °C; 200 rpm) in 3 ml LB broth also containing Kanamycin (100 μg/ml).

The plasmids with the A/B standards were then extracted and purified using the ZymoPURE Plasmid Miniprep Kit (Zymo Research), according to the manufacturer’s protocol. Purified plasmids were linearized by overnight digestion (37 °C) with EcoRI-HF (NEB) and the products were then visualized on 1% agarose gel. The linear A/B standards were finally treated with Proteinase K and further purified with the Zymo ChIP DCC-25 purification kit (Zymo Research). The final A/B standards were quantified using Qubit dsDNA HS Assay on Qubit 2.0 Fluorometer (Life Technologies) and verified on the Agilent TapeStation with the High Sensitivity DNA Screen Tape Analysis (Agilent Technologies).

### In vitro transcription

The ChIP purified A/B standards were submitted to an in vitro transcription reaction, incubated overnight at 37 °C, using the MEGAscript T7 Transcription kit (ThermoFisher) according to the manufacturer’s protocol. The resulting product was then treated with Turbo DNase and the remaining RNA was purified with the Zymo RCC-25 column purification-25 kit (Zymo Research). The A/B RNA standards were quantified using Qubit RNA HS Assay on Qubit 2.0 Fluorometer (Life Technologies) and then verified on the Agilent TapeStation (Agilent Technologies) with the RNA ScreenTape Analysis (Agilent Technologies).

### Covid-19 patient samples

Patient samples that had been previously diagnosed with COVID-19 that were used for validation tests were collected at the SAViD laboratories at Randwick, Sydney as part of a quality assurance study. The samples constitute viral RNA extracts (using the Roche MagNA Pure extraction kit) on nasopharyngeal swabs from patients testing positive for SARS-CoV-2 infection. cDNA was generated from the RNA extracts isolates using Thermo Fisher Superscript IV VILO Master Mix, according to the manufacturer’s protocol. cDNA was amplified with each of 14 × ~ 2.5 kb amplicons tiling the SARS-CoV-2 genome, according to a custom protocol^11^. Amplicons were then cleaned with AMPure beads and pooled at equal abundance.

### Commercial controls

We acquired control plasmids from IDT Technologies to be used in the CDC real-time RT-PCR diagnostic assay of SARS-CoV-2. The positive control (2019-nCoV_N_Positive Control, catalog number: 10006770) contained the complete nucleocapsid gene sequence, whilst the negative control contains a portion of the human *RPP30* gene sequence. The stocks for each of the plasmids were delivered at 200,000 copies/μl in IDTE pH 8.0. For the real-time RT-PCR, plasmids were diluted to 4,000 copies/μl each.

### Quantitative real time PCR

Twenty μl reactions were prepared containing 5 μl of input RNA (patient samples, A/B standards or IDT controls), 5 μl of TaqPath 1-Step RT-qPCR Master Mix, 1.5 μl of the combined CDC primers/probe set and 8.5 μl of Nuclease-free water.

The experiment was performed on QuantStudio 7 Flex real-time PCR systems (Thermo Fisher). Thermo cycling was performed at 25 °C for 2 min to allow UNG incubation, followed by 15 min at 50 °C for reverse transcription, then 2 min at 95 °C for enzyme activation and finally 45 amplification cycles at 95 °C for 3 s and 55 °C for 30 s.

### Dilution series

We performed two different serial dilution experiments with the chimeric A/B standards. The first (Fig. 2a) was a tenfold serial dilution of standards A and B alone, to test their performance in the real-time RT-PCR assay. The baseline concentration was 3.96 × 10^8^ copies/μl for standard A and 4.22 × 10^8^ copies/μl for standard B. We diluted each standard until 10^–5^.

The second serial dilution (Fig. 2b) was to estimate the limit of detection (LoD) for the chimeric A/B standards. For this experiment, standards A and B were individually spiked into universal human RNA samples. The baseline concentration was 3964 copies/μl for standard A and 4221 copies/μl for standard B and they were each added into 100 ng of universal human RNA. We made 100-fold dilutions for the A/B standards until 10^–6^, and the experiment was performed in three technical replicates.

### Ethics declarations

All procedures involving human participants were in accordance with the ethical standards of the institutional and/or national research committee and with the 1964 Helsinki Declaration and its later amendments or comparable ethical standards. The procedures were approved by the Human Research Ethics Committee at South Eastern Sydney Local Health District (SESLHD), under approval 2020/ETH00287. Where relevant, informed consent was obtained from individual research participants.

## Supplementary Information

Supplementary Tables.

Supplementary Figure.
