# Supplementary material for: Chimeric synthetic reference standards enable cross-validation of positive and negative controls in SARS-CoV-2 molecular tests
Source: Sci Rep. 2021 Jan 29;11:2636. doi: 10.1038/s41598-021-81760-0 (PMC7846570; doi:10.1038/s41598-021-81760-0)
Supplement: Supplementary file 2 — Supplementary Figure. [file 41598_2021_81760_MOESM2_ESM.pdf]

Supplementary Materials:

Chimeric synthetic reference standards enable cross-validation of positive and negative controls in SARS-CoV-2 molecular tests.

Bindu Swapna Madala, Andre L. M. Reis, Ira W. Deveson, William Rawlinson & Tim R. Mercer

TABLE OF CONTENTS

Supplementary Figure 1. **P2**

APPENDIX

Supplementary Table 1 -WHO endorsed real-time RT-PCR prime/probe sets per country.

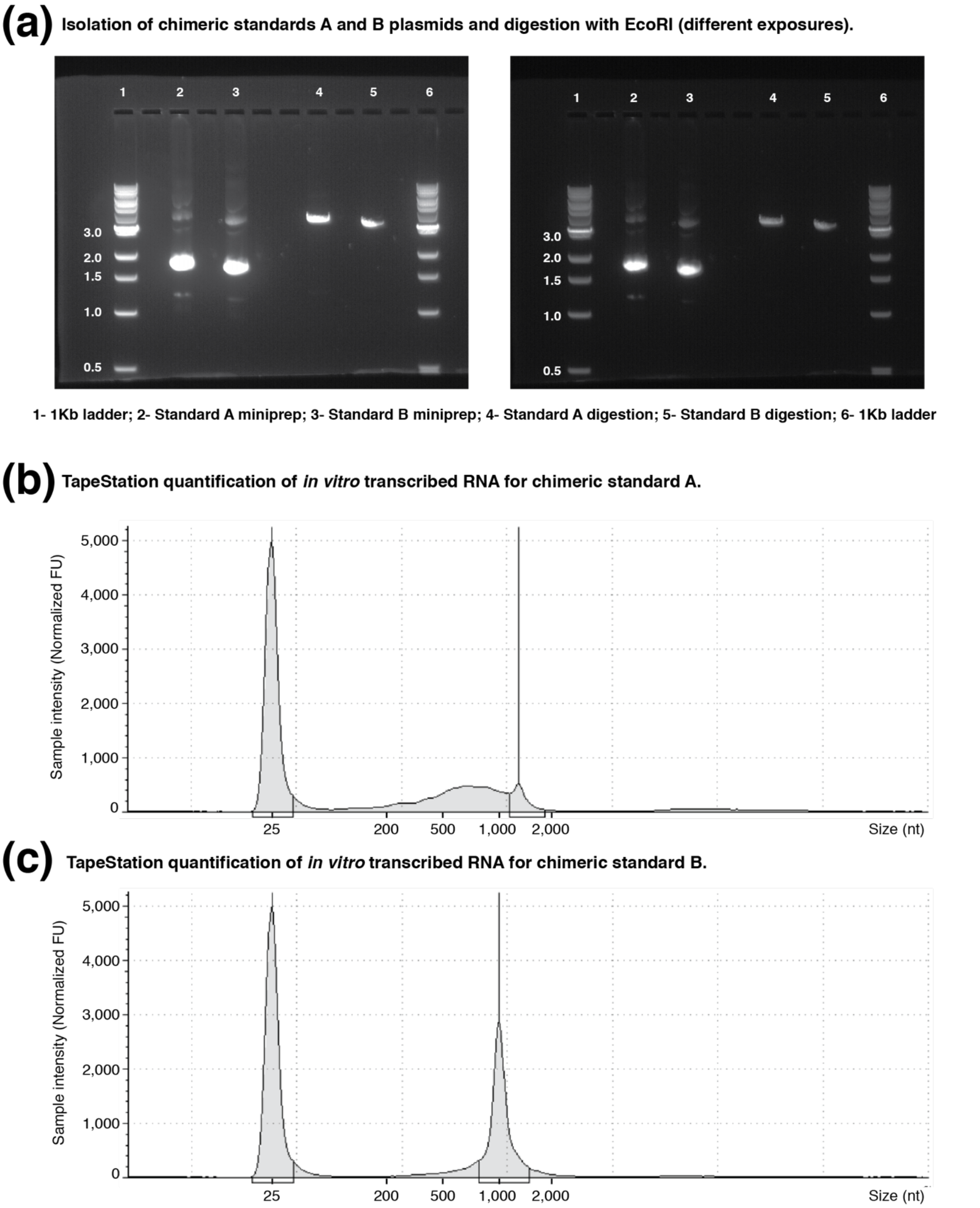

Supplementary Figure 1. (**a**) Gel electrophoresis showing miniprep results for chimeric A/B standards and digestion with EcoRI restriction enzyme. Those are photos of the same gel at different exposures. (**b**) TapeStation results for the purified RNA product, resulting from the *in vitro* transcription of the linearized standard A. (**c**) TapeStation results for the purified RNA product, resulting from the *in vitro* transcription of the linearized standard B.
